# Anti-Obesity Effects of Matoa (*Pometia pinnata*) Fruit Peel Powder in High-Fat Diet-Fed Rats

**DOI:** 10.3390/molecules26216733

**Published:** 2021-11-07

**Authors:** Toshikazu Suzuki, Mayumi Nagata, Natsuko Kagawa, Shiori Takano, Nahrowi, Jun Nomura

**Affiliations:** 1 Department of Health and Nutrition, Wayo Women’s University, 2-3-1 Konodai, Ichikawa, Chiba 272-8533, Japan; k11.clover13@gmail.com (M.N.); s-takano@wayo.ac.jp (S.T.); 2 Center for Environment, Health and Field Sciences, Chiba University, 6-2-1 Kashiwa-no-ha, Kashiwa, Chiba 277-0882, Japan; knatsuko@faculty.chiba-u.jp; 3 Department of Nutrition and Feed Technology, Faculty of Animal Science, IPB University, Bogor 16680, Indonesia; nahrowi@apps.ipb.ac.id; 4 Department of School Health Nursing, Faculty of Education, Chiba University, 1-33 Yayoicho, Inage-ku, Chiba 263-8522, Japan

**Keywords:** ApoB-48, Caco-2 cell monolayers, hederagenin saponin, hepatic lipid accumulation, high-fat diet, obesity, *Pometia pinnata*

## Abstract

Fruit peels, pericarps, or rinds are rich in phenolic/polyphenolic compounds with antioxidant properties and potentially beneficial effects against obesity and obesity-related non-communicable diseases. This study investigated the anti-obesity effects of matoa (*Pometia pinnata*) and salak (*Salacca zalacca*) fruit peel. Neither matoa peel powder (MPP) nor salak peel powder (SPP) affected the body weight, visceral fat weight, or serum glucose or lipid levels of Sprague–Dawley rats when included as 1% (*w*/*w*) of a high-fat diet (HFD). However, MPP significantly decreased the hepatic lipid level. MPP at a dose of 3% (*w*/*w*) of the HFD decreased body weight, visceral fat, and serum triglyceride levels as well as the hepatic lipid content. The inhibitory effect of MPP on hepatic lipid accumulation was not enhanced when its concentration was increased from 1% to 3% of the HFD. The anti-obesity effect of matoa was partly explained by the inhibitory effect of the matoa peel extract on fatty acid-induced secretion of ApoB-48 protein, a marker of intestinal chylomicrons, in differentiated Caco-2 cell monolayers. We identified hederagenin saponins that are abundant in MPP as potential anti-obesity substances. These results will contribute towards the development of functional foods with anti-obesity effects using the matoa fruit peel.

## 1. Introduction

Overweight/obesity is defined as abnormal body weight (BW) with excessive fat accumulation caused by an imbalance between energy intake and consumption. A body mass index of 25–30 and ≥30 indicates overweight and obesity, respectively. Globally, 26% and 13% of adults aged 18 years and older in 2016 were overweight and obese, respectively [1,2]. The prevalence of obesity is increasing and has nearly tripled since 1975. Obesity is a risk factor for non-communicable diseases, including type 2 diabetes, hypertension, heart disease, stroke, non-alcoholic steatohepatitis, osteoarthritis, and several types of cancer [2,3]. According to the Global Burden of Disease study, the number of premature deaths attributed to obesity increased from 2.2 million in 1990 to 4.7 million in 2017 [4]. To develop safe and reliable treatment, scientists and researchers have focused on discovering anti-obesity agents with minimal adverse effects over the last two and a half decades; these agents include phytochemicals from plant-derived foods and herbs [3,5,6]. Various phytochemicals such as flavonoids, phenols/polyphenols, terpenoids, alkaloids, and saponins have demonstrated anti-obesity bioactivity [6].

Epidemiological research has shown that most fruits exert anti-obesity effects despite containing large amounts of simple sugars. This is probably attributable to their micronutrient and non-essential phytochemical content, including phenolic compounds that modulate various physiological cascades such as oxidative stress reduction pathways [7]. Besides those in the pulp of fruits, beneficial bioactive compounds are also found in the peels, pericarps, rinds, and seeds; these are by-products that are now considered unconventional sources of innovative functional foods [8]. For example, John et al. reported the anti-obesity effects of powdered rinds of yellow (*Garcinia dulcis*) and purple mangosteen (*Garcinia mangostana*) [9,10].

*Pometia pinnata* (family Sapindaceae), a fruit tree species known as matoa in Indonesian or Fijian longan in English, is distributed throughout the Asia-Pacific, including Indonesia, Tonga, Fiji, Malaysia, Thailand, and Sri Lanka. The fleshy fruit of *P. pinnata* is edible and valued as a traditional medicine for the treatment of hypertension as well as obstetric, gynecological, and abdominal ailments, including stomach complaints, diarrhea, and dysentery [11]. Matoa by-products such as the leaves, seeds, fruit peels, and stem bark are inedible but have potential bioactivities, including antioxidant, antimicrobial, and antidiabetic activities [12]. In terms of antidiabetic properties, a study reported on the inhibitory activity of α-glucosidase in the ethanol extract of matoa stem bark [13]. To the best of our knowledge, there have been no in vivo or in vitro studies on the anti-obesity effects of matoa or its various derived products.

Previously, we evaluated the effect of simulated in vitro digestion on the antioxidant activities of seed and peel extract of six different tropical fruits from Indonesia [14]. Among the fruit by-product samples we investigated, the aqueous supernatant of matoa peel powder (MPP) had the highest total phenolic content and 1,1-diphenyl-2-picrylhydrazyl (DPPH) radical scavenging activity and the strongest inhibitory effect on lipid peroxidation after undergoing in vitro digestion. In contrast, the aqueous acetonitrile extract of salak (*Salacca zalacca*) peel powder (SPP) had the highest DPPH radical scavenging activity and total phenolic content before in vitro digestion. Furthermore, in vitro digestion reduced the radical scavenging activity of the salak peel extract to less than 40% of its pre-digestion level, suggesting that matoa peel may be more suitable for use in functional foods or dietary supplements than salak peel.

This study investigated the effects of matoa peel and salak peel on serum parameters, hepatic lipid levels, weight gain, and organ weights, including visceral fat weight, in high-fat diet (HFD)-fed rats. We also examined the effect of matoa peel extracts using differentiated Caco-2 cell monolayers to monitor basolateral secretion of ApoB-48—a suitable model system for studying the effect of bioactive compounds on the formation of fatty acid-dependent chylomicrons in the intestine [15,16] and HuH-7 hepatoma cells—an in vitro model system for studying the effect of bioactive compounds on the formation of liver steatosis [17]—to investigate the mechanism of the aforementioned in vivo effects of MPP on HFD-induced obesity. Moreover, we partially characterized and compared the chemical composition of matoa peel and salak peel. Finally, we discuss the possible mechanism underlying the anti-obesity effect of matoa peel.

## 2. Results

### 2.1. Biological Effects

#### 2.1.1. Comparison of the Effects of MPP and SPP in HFD-Fed Rats (Animal Experiment 1)

After four weeks of dietary intervention receiving the controlled diet as described in the Materials and Methods section (see Table 6), the average daily intake did not differ among the four treatment groups of rats (Table 1). The final body, liver, peritesticular fat, perirenal fat, and mesenteric fat weights were higher in the HFD-group (HF) than in the normal diet group (N), demonstrating HFD-induced obesity. The addition of either 1% MPP (1M group) or 1% SPP (1S group) to the HFD did not significantly affect any of the aforementioned weight parameters when compared with the parameters of the HF group. Furthermore, the liver, perirenal fat, and mesenteric fat weights in the 1M group and the perirenal fat weight in the 1S group were not significantly different from those in the N group. There were no differences in serum glucose and lipid levels among the four groups (Table 2). The serum activity of hepatic enzymes was unchanged in both the 1M and 1S groups, suggesting that the fruit peel at a dose of 1% of the HFD was not hepatotoxic.

**Figure 1:**
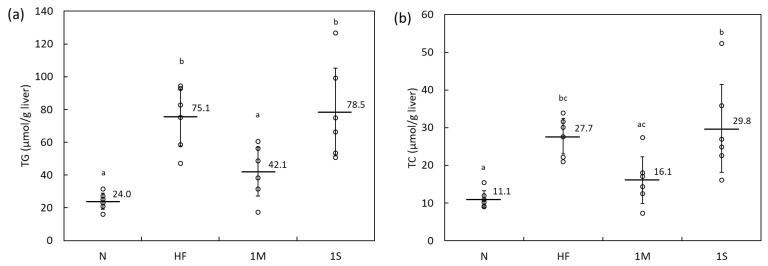
(**a**) Hepatic triglyceride and (**b**) total cholesterol accumulation in Sprague–Dawley rats fed a high-fat diet containing 1% matoa peel powder (MPP) or salak peel powder (SPP) for four weeks (Animal Experiment 1). N, normal diet; HF, high-fat diet; 1M, high-fat diet containing 1% MPP; 1S, high-fat diet containing 1% SPP. Data are shown as dot plots with means ± standard deviations (*n* = 6). Means with different letters differ significantly (*p* < 0.05).

Nevertheless, 1% MPP decreased hepatic lipid levels in HFD-fed rats (Figure 1). In particular, hepatic triacylglycerol (TG) levels decreased significantly in the 1M group compared to those in the HF group (Figure 1a). Conversely, hepatic TG and total cholesterol (TC) levels of the HF and 1S groups were almost the same (Figure 1), suggesting that MPP may exert some anti-obesity effects when included in an HFD, but SPP would not.

#### 2.1.2. Dose-Dependency of Anti-Obesity Effects of MPP in HFD-Fed Rats (Animal Experiment 2)

**Figure 2:**
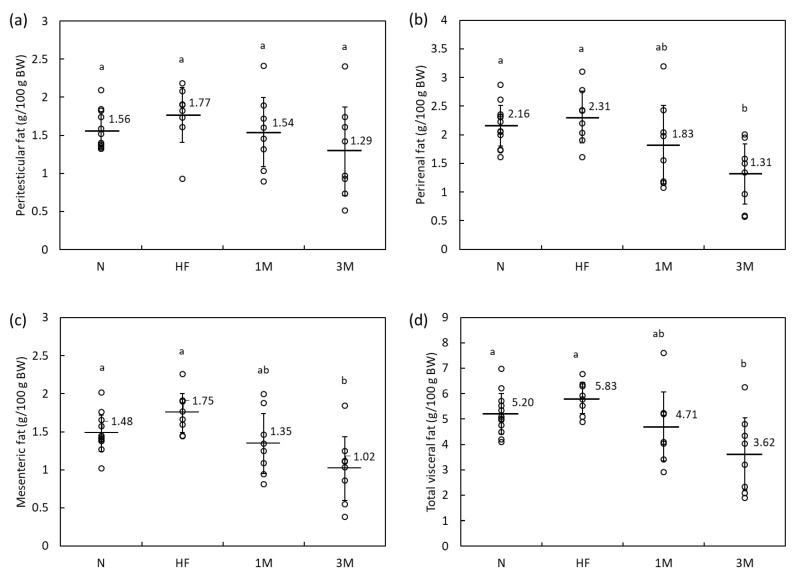
Relative weight expressed as g per 100 g BW of (**a**) peritesticular, (**b**) perirenal, (**c**) mesenteric, and (**d**) total visceral fat in rats fed a high-fat diet containing 1% or 3% matoa peel powder (MPP) for four weeks (Animal Experiment 2). The total visceral fat weight was calculated by summing the peritesticular, perirenal, and mesenteric fat weights. Data are shown as dot plots with means ± standard deviations; N, normal diet (*n* = 12); HF, high-fat diet (*n* = 8); 1M, high-fat diet containing 1% MPP (*n* = 8); 3M, high-fat diet containing 3% MPP (*n* = 8). Means with different letters differ significantly (*p* < 0.05).

The reproducibility and dose-dependency of the anti-obesity effects of MPP were assessed by feeding the rats an HFD containing 1% or 3% MPP for four weeks (group 1M and 3M, respectively). The average dietary intake was slightly lower in the HF group than in the N group (Table 3). Additionally, dietary MPP resulted in mild dose-dependent decreases in food intake, BW, liver weight, perirenal fat weight, and mesenteric fat weight when compared with those in the HF group (Table 3). Body fat was assessed by comparing peritesticular, perirenal, and mesenteric fat, and the sum of the three visceral fat weights per 100 g BW (Figure 2). We found that the fat weights per 100 g BW were slightly higher in the HF group than that in the N group. The total visceral fat weight was calculated by summing the weights of the peritesticular fat, perirenal fat, and mesenteric fat (expressed on a g per 100 g BW basis). The HF group had slightly increased total visceral fat compared with the N group, although the difference was not statistically significant (Figure 2d). Dietary MPP resulted in a moderate decrease in fat weight in a dose-dependent manner; the perirenal fat, mesenteric fat, and total visceral fat weight were significantly lower in the 3M group than in the HF group, suggesting an anti-obesity effect of MPP at this dose.

**Figure 3:**
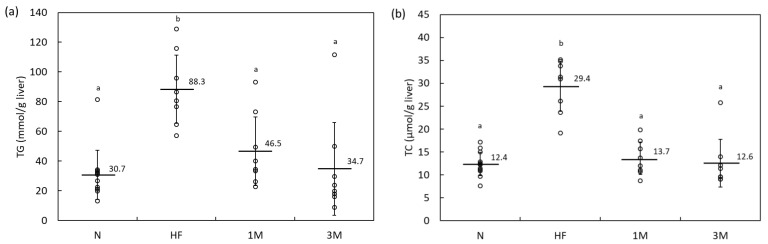
Accumulation of (**a**) hepatic triacylglycerol (TG) and (**b**) total cholesterol (TC) in Sprague–Dawley rats fed a high-fat diet containing 1% or 3% matoa peel powder (MPP) for four weeks (Experiment 2). N, normal diet (*n* = 12); HF, high-fat diet (*n* = 8); 1M, high-fat diet containing 1% MPP (*n* = 8); 3M, high-fat diet containing 3% MPP (*n* = 8). Data are shown as dot plots with means ± standard deviations. Means with different letters differ significantly (*p* < 0.05).

The serum biochemical parameters and hepatic lipid level analyses also provided evidence for the anti-obesity effect of MPP. A dose-dependent decrease in serum TG and a slight but insignificant increase in serum high-density lipoprotein cholesterol (HDL-C) levels were observed when MPP was added to the HFD (Table 4). Accumulation of hepatic TG and TC caused by the HFD was strongly inhibited by the addition of MPP (Figure 3). The inhibitory effect of MPP on hepatic lipid accumulation seemed stronger than its effect on serum lipid levels, as the hepatic lipid levels in the 1M group were closer to those of the 3M group than to those of the HF group. Conversely, residual fecal TG levels were similar among the HF, 1M, and 3M groups (Table 3). Non-hepatotoxicity of MPP at up to 3% of the HFD was confirmed by the lack of increase in serum alanine transaminase (ALT), aspartate transaminase (AST), or gamma-glutamyl transpeptidase (γ-GTP) levels in the 3M group (Table 4).

#### 2.1.3. Matoa Peel Extract Suppressed Lipid Micelle-Dependent ApoB-48 Secretion in Caco-2 Monolayers

**Figure 4:**
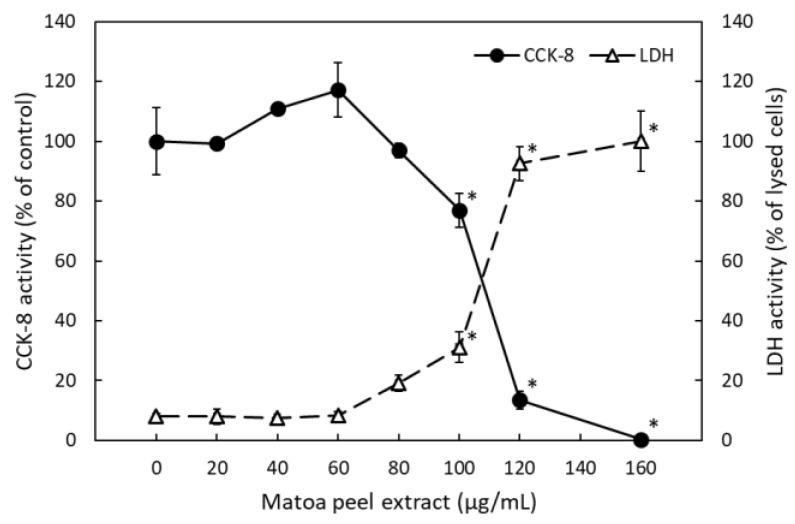
Effect of matoa peel extract (0–160 µg/mL) on Caco-2 cells. Cell viability (filled circle) was determined using a commercial cell counting kit (CCK-8 activity; expressed relative to the activity of untreated cells with 100% activity). Cell toxicity (unfilled triangle) was determined using a lactate dehydrogenase (LDH) assay in the culture medium and expressed relative to a detergent-lysed culture representing 100% toxicity. Data are presented as means ± standard deviations (*n* = 3). * *p* < 0.05 vs. untreated cells.

We believe that the anti-obesity effect of MPP may be partly attributed to compounds in the matoa peel that have an inhibitory effect on intestinal fatty acid absorption; a Caco-2 monolayer system was used to investigate this hypothesis. Basolateral secretion of ApoB-48-containing lipoprotein was induced by applying complex lipid micelles to the apical side of the monolayers, mimicking intestinal absorption of fatty acids and lipids [18]. We first performed a cell growth and cytotoxicity assay to establish the non-toxic concentration of the matoa peel extract (Figure 4). The cell growth evaluation using a commercial cell counting kit and the cytotoxicity evaluation using a lactate dehydrogenase (LDH) assay revealed that more than 100 µg/mL of matoa peel extract was toxic to Caco-2 cells. At 80 µg/mL, significant toxicity was not observed; however, LDH activity was mildly but insignificantly increased. Therefore, we used matoa peel extract at a concentration of no more than 60 µg/mL to determine basolateral ApoB-48 secretion.

**Figure 5:**
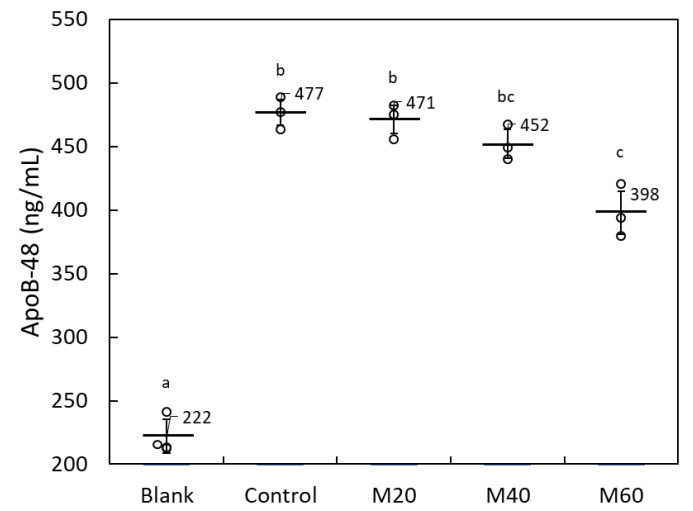
Dose-dependent inhibitory effect of matoa peel extract on the basolateral secretion of ApoB-48 protein in Caco-2 monolayers. The blank group represents cells to which no lipid micelle or matoa extract was applied; the control group cells were treated with lipid micelles without matoa extract; M20, M40, and M60 indicate cells treated with lipid micelles containing matoa extract at 20, 40, or 60 µg/mL, respectively. Data are shown as dot plots with means ± standard deviations (*n* = 3). Means with different letters differ significantly (*p* < 0.05).

The concentration of ApoB-48 in the basolateral well medium increased by 255 ng/mL when lipid micelles were added to the apical medium, demonstrating micelle-mediated ApoB-48 secretion (blank vs. control; Figure 5). In the presence of matoa peel extract with micelles on the apical side, basolateral ApoB-48 secretion decreased mildly and in a dose-dependent manner (M20 > M40 > M60; Figure 5). At 60 µg/mL, micelle-mediated ApoB-48 secretion decreased by 31% when compared with the control group. These results imply that the anti-obesity effect of MPP is at least partly mediated by the inhibition of intestinal lipid absorption by the compound(s) contained in the matoa peel.

#### 2.1.4. Matoa Peel Extract Did Not Suppress Oleic Acid-Dependent Lipid Accumulation in HuH-7 Hepatoma Cells

We observed decreased hepatic lipid accumulation by MPP in HFD-fed rats (Figure 1 and Figure 3), suggesting that compounds in matoa peel might directly inhibit lipid accumulation. HuH-7 hepatoma cells, an in vitro model for fatty liver [17], were used to determine whether the matoa peel extract could inhibit fatty acid-induced hepatic lipid accumulation (Figure S1). Cell growth and cytotoxicity evaluation using a cell-counting reagent and LDH assay revealed that up to 31 µg/mL of matoa peel extract was non-toxic to HuH-7 cells (Figure S1a). Then, HuH-7 cells were exposed to 0.5 mM oleic acid (OA) for 24 h to measure the effect of matoa peel extract on hepatic lipid accumulation in vitro (Figure S1b). Compared to the control-treated cells (Figure S1b1), an increase in Oil Red O-stained lipid droplets was observed in OA-treated cells (Figure S1b2). However, matoa peel extract at 30 µg/mL did not alleviate OA-induced lipid droplets (Figure S1b4). This result suggests that the compounds in the MPP do not affect hepatic lipogenesis or lipolysis in vivo.

### 2.2. Chemical Analyses

#### 2.2.1. Identification of Saponin in Matoa Peel

The chemical analysis of MPP was conducted using the matoa extract. From a separated fraction that was soluble in 50% (*v*/*v*) aqueous methanol, compound **1** was isolated at a yield of approximately 0.4% (*w*/*w* of dried peel). The nuclear magnetic resonance (NMR) spectrum of compound **1** showed a triterpene saponin composed of an aglycone moiety and a sugar moiety. Comparison of the spectra of compound **1** with those of saponins reported in the literature [19] identified the saponin as 3-*O*-α-L-arabinofuranosyl(1→3)-α-L-rhamnopyranosyl(1→2)-α-L-arabinopyranoside of hederagenin (Figure S2).

#### 2.2.2. Hederagenin Saponin (HGS) Content in Matoa and Salak Peels

Acid hydrolysis removes the sugar moiety from saponins with an aglycone moiety consisting of hederagenin, thus producing sugar-free hederagenin molecules. Therefore, the HGS content of matoa and salak peels could be determined after applying hydrochloric acid treatment and subsequently extracting with chloroform to obtain sugar-free hederagenin. When the standard solution of hederagenin (0.96 µg/mL in methanol) was subjected to this method, the recovery was 65%. Hydrolysis of the peel extract with water followed by the same chloroform extraction method was performed to serve as the control and to obtain the background spectrum of sugar-free hederagenin. Hederagenin concentrations were measured by liquid chromatography-mass spectrometry (LC-MS), and changes in the hederagenin concentration of the extracts were calculated by subtracting the mean of the control measurements (*n* = 3) from each measurement of the acid hydrolyzed samples. The HGS content in the matoa and salak peel powder were 1.41 and 0.0154% (*w*/*w*), respectively (Table 5). The HGS content was more than 90-fold higher in matoa than in salak peel; this finding implies that HGS may be one of the candidate compounds involved in the anti-obesity effect of MPP in HFD-fed rats.

## 3. Discussion

Many researchers have explored and exploited the anti-obesity effects of natural compounds derived from foods and herbs [3]. Phytochemicals, including phenolic compounds, alkaloids, triterpenoids, and saponins, have been identified as natural anti-obesity agents. The inedible waste of plant fruits such as peels, pericarps, rinds, and seeds are phytochemical-rich raw materials with potential anti-obesity effects [9,10,20,21,22]. The present study investigated the anti-obesity effect of two tropical fruit peels with high total phenolic content in HFD-fed rats and revealed that matoa peel exerted an anti-obesity effect whereas salak peel did not. MPP at 1% significantly reduced hepatic TG and TC contents in HFD-fed rats. We observed dose-dependent decreases in BW, liver, and visceral fat weights, and serum TG levels in HFD-fed rats that received MPP as part of the HFD. The decrease in hepatic TG and TC contents observed in the MPP-treated groups seemed to reach a plateau at 1% MPP content in the HFD, as the observed effects were similar between 1% M and 3% M. Furthermore, analysis of serum hepatic enzyme activities showed that MPP showed no hepatotoxicity at the highest tested concentration of 3%. These results demonstrate that MPP exhibits anti-obesity activity and may be useful as a food ingredient in controlled anti-obesity diets.

We also investigated the possible biological mechanisms and active components involved in the anti-obesity effect of the methanolic extracts of the fruit peels. In Caco-2 monolayers, matoa peel extracts decreased lipid micelle-dependent ApoB-48 secretion to the basolateral side in a dose-dependent manner. Additionally, we identified relatively high levels of the natural compound and potent candidate anti-obesity agent HGS **1** in matoa peel but not in salak peel. HGS **1** has already been isolated from matoa leaves [19], which also contain another type of HGS, 3-*O*-[α-L-arabinofuranosyl(1→4)–α-L-rhamnopyranosyl(1→2)-α-L-arabinopyranosyl]hederagenin [23]. Matoa (*P. pinnata*) is a large evergreen tree of the plant family Sapindaceae. The fruit peel of another member of this family, *Sapindus mukorossi,* also contains HGSs [24]. The anti-tumor and anti-neutrophil activating activities of HGSs have been reported [25,26,27], but their anti-obesity activity has not been reported. However, other triterpenoid saponins in foods and herbs have been shown to modulate metabolic pathways and thereby prevent obesity [28,29]. Recently, Tsai et al. reported the anti-obesity effect of soyasaponins in HFD-fed C57BL/6J mice [30]. In addition, Wu et al. demonstrated that oleanane and ursane-type triterpenoids isolated from *Cyclocarya paliurus* (CP) downregulated intestinal ApoB-48 secretion and that a hydroxy group at C-23 in the triterpenoid structure seemed to be essential for their activities [16]. These results may be related to the anti-hyperlipidemic effect of CP ethanolic extract in HFD-fed Kunming mice [31]. HGS **1** and soyasaponins have oleanane-type triterpenoid aglycone moieties with a hydroxy group at C-23. Furthermore, hederagenin, the aglycone moiety of HGS, exhibited multiple anti-atherosclerotic activities in rats, including improved serum lipid profiles without hepatic toxicity when administered at 20 mg/kg/day [32]. This dose of hederagenin is equivalent to 20 g of the feed given to the 3M group in the present study (Animal Experiment 2; Section 2.2.1); the calculation was based on the dietary composition data for rats weighing 230 g each. In summary, the anti-obesity effect of MPP may be, in part, attributable to the inhibitory effect of HGSs on the intestinal absorption of lipids in digestive micelles.

Dietary saponins, such as diosgenin, morgoside, sessiloside, sibutramine, and soyasaponin, have the potential to prevent obesity [28]. These saponins can suppress weight gain in mice fed an HFD, in addition to decreasing the visceral adipose tissue mass and the lipid levels in the serum and liver. The inhibitory activity of saponins against pancreatic lipase is considered one of the mechanisms underlying their anti-obesity effect [28]; however, in this study, addition of MPP to the HFD did not increase the fecal TG content, suggesting that pancreatic lipase activity was not inhibited by the compounds in MPP. Similarly, the anti-obesity effects of soyasaponins and fermented soymilk were not associated with increased fecal lipid levels [30,33]. Moreover, soyasaponins did not inhibit pancreatic lipase in vitro, indicating that pancreatic lipase inhibition is not always involved in the anti-obesity effect of bioactive foods.

Some other well-described mechanisms underlying the anti-obesity activity of saponins include adipogenesis inhibition and lipogenesis activation in adipocytes and hepatocytes, as demonstrated in experiments using cultured cells [28]. However, we failed to confirm the anti-obesity effects of matoa peel extract at non-toxic concentrations in HuH-7 hepatoma cells (Figure S1). Because the methanolic extract of matoa peel contains multiple phenolic compounds, some toxic compounds may impede the detection of adipogenesis/lipogenesis in cultured cells. Furthermore, the absorption rate of saponins in the human gastrointestinal tract is low. Some saponins are converted to more bioavailable and bioaccessible compounds, such as sapogenins (aglycones of saponins), by the colonic microbiota [34]. The inhibitory effects of soyasapogenols, the aglycones of soyasaponins, have been demonstrated in 3T3-L1 preadipocytes [30]. Therefore, it is possible that hederagenin may have reached the liver or adipose tissue while HGS **1** did not. Therefore, the use of hederagenin may be more suitable than matoa peel extracts/HGSs for investigating the effect on adipogenesis and lipogenesis in cell culture systems.

This study has some limitations. First, we cannot rule out the involvement of compounds other than HGS in the anti-obesity effect of MPP in HFD-fed rats. The content of these compounds in matoa and salak peels may differ significantly. Therefore, future research should focus on separating and identifying compounds in fruit peel extracts using organic solvents, identifying the candidate compounds by comparing the fruit peel chemical compositions, and investigating their anti-obesity bioactivities. Second, we did not thoroughly evaluate the safety of MPP as a food. The matoa fruit peel is usually not consumed or used for medicinal purposes by the local people of Indonesia. Therefore, its safety as a food ingredient cannot be assumed. Although we did not observe hepatotoxicity in rats fed an HFD containing 3% MPP for four weeks, we have not demonstrated the food safety of MPP. Furthermore, a methanolic extract of matoa peel previously showed toxic effects in a brine shrimp lethality test (LC_50_ = 139.41 ppm) [12]. Therefore, chronic and sub-chronic toxicity, reproductive toxicity, genotoxicity, and carcinogenicity tests should be performed before proposing MPP as a healthful supplement or food ingredient.

## 4. Materials and Methods

### 4.1. Plant Material

#### 4.1.1. Fruit Peel Powders

Matoa and salak fruits were obtained from local markets in Bogor, Indonesia. The peels were separated from the fruits and frozen at −20 °C, followed by freeze-drying and grinding into powder. The peel powders were stored at 4 °C until further use. The compositional analyses of MPP and SPP were performed at Institut Pertanian Bogor (Fakultas Petrnakan; Bogor, Indonesia). The results (expressed as %) for MPP and SPP were as follows: dried matter, 95.53 and 95.69; ash, 5.06 and 5.06; crude protein, 6.79 and 6.03; crude fiber, 9.03 and 34.60; extract ether, 1.14 and 0.02; nitrogen-free extract, 73.51 and 49.98, respectively.

#### 4.1.2. Matoa Peel Extract (Ethanol Extraction)

MPP (11.0 g) was steeped in ethanol (300 mL) for four days at 4 °C, sonicated for 30 min, and then filtered through Celite No. 503 (FUJIFILM Wako Pure Chemical Corp., Osaka, Japan). The filtrate was concentrated to a total volume of 25 mL that was subsequently filtered through a 0.45 μm nylon membrane (Shimadzu GLC Ltd., Tokyo, Japan), evaporated under reduced pressure, and dried in vacuo to obtain a yellow-green crude oil (0.575 g). The extracts were stored at 4 °C until use.

### 4.2. Biological Effects

#### 4.2.1. Animal Experiments

The experimental protocol for the care and use of the animals was conducted according to the ethical norms approved by the Animal Care and Use Committee of Wayo Women’s University (Chiba, Japan). The approval numbers were 1801 for Animal Experiment 1 (approved on 11 September 2018) and 1901 for Animal Experiment 2 (approved on 19 August 2019). Six-week-old male Sprague–Dawley rats were purchased from CLEA Japan, Inc. (Tokyo, Japan). The animals were housed individually in a metabolic cage at 25 ± 2 °C and 50–60% humidity with a 12 h light-dark cycle and had free access to food and water. They were initially acclimated by feeding on a standard chow diet for seven days. Subsequently, the rats (average weight in Animal Experiment 1 = 249.3 ± 6.2 g; Animal Experiment 2 = 224.7 ± 7.6 g) were divided into four groups, performed the dietary intervention for four weeks, and weighed every week. In Animal Experiment 1, the first group (N; *n* = 6) was fed a normal diet (approximately 10% calories from fat), the second group (HF; *n* = 6) was fed an HFD (approximately 40% calories from fat), the third group (1M; *n* = 6) was fed an HFD containing 1% MPP (10 g/kg diet), and the fourth group (1S; *n* = 6) was fed an HFD containing 1% SPP (10 g/kg diet).

In Animal Experiment 2, the first group (N; *n* = 12) was fed a normal diet, the second group (HF, *n* = 8) was fed an HFD, the third group (1M; *n* = 8) was fed an HFD containing 1% MPP (10 g/kg diet), and the fourth group (3M; *n* = 8) was fed an HFD containing 3% MPP (30 g/kg diet). The basal components of the experimental diets were based on the AIN-93G (American Institute of Nutrition) formulation [35], and its composition is shown in Table 6. Daily food intake was monitored by weighing the amount of initial and leftover food in a 24 h interval and subtracting the leftover from the initial weight. The energy intake during the experiment was subsequently calculated based on the daily food intake data.

#### 4.2.2. Collection of Serum and Organ Samples

After four weeks on the controlled diets, the rats were anesthetized with isoflurane vapor after overnight deprivation. Blood samples were collected from the abdominal aorta, and the rats were euthanized by exsanguination. Serum was isolated by centrifugation at 1700 × *g* for 10 min at 4 °C. The liver, kidneys, spleen, peritesticular fat, perirenal fat, and mesenteric fat were excised and weighed. Serum and liver samples were flash-frozen and stored at −80 °C until use.

#### 4.2.3. Serum Biochemical Analyses

The levels of glucose, TG, TC, HDL-C, ALT, AST, and γ-GTP in the serum were determined using a DRI-CHEM 4000 Chemistry Analyzer (Fujifilm Holdings, Tokyo, Japan).

#### 4.2.4. Determination of Hepatic Lipid Content

Total lipids were extracted from the liver samples using chloroform-methanol (2:1) [36]. First, the lipid-containing organic phase was placed in a glass tube in a dark draft chamber for two days to evaporate the solvent and then stored at −80 °C until use. The remaining lipid pellet was dissolved in 2-propanol-Triton X-100 (9:1) with sonication for 5 min, and TG and TC levels were determined using a Triglyceride E-Test (FUJIFILM Wako Pure Chemical Corp.) and Total Cholesterol E-Test (FUJIFILM Wako Pure Chemical Corp.), respectively.

#### 4.2.5. Determination of Fecal TG Content

Feces were collected for three days during the final week of the feeding experiment and stored at −80 °C until analysis. After lyophilizing and weighing the dry feces, they were powdered using a mortar and pestle for lipid extraction. Lipid extraction from feces was performed according to the method described by Kraus et al. [37]. After evaporating the organic solvent, the resulting lipids were resuspended in 2-propanol-Triton X-100 (9:1) and analyzed using the Triglyceride E-Test (FUJIFILM Wako Pure Chemical Corp.).

#### 4.2.6. Cell Culture

The human colonic adenocarcinoma cell line (Caco-2; RCB0988) at passage no. 46 and the human hepatoma cell line (HuH-7; JCRB0403) at passage no. 49 were obtained from the RIKEN BioResource Center (Tsukuba, Japan) and the Japanese Collection of Research Bioresources Cell Bank (Osaka, Japan). Cells at passage numbers between 50 and 54 of Caco-2 and between 56 and 60 of HuH-7 were used for the experiments. Caco-2 cells were cultured in Dulbecco’s Modified Eagle Medium (DMEM) with 4.5 g/L glucose (FUJIFILM Wako Pure Chemical Corp.) containing 10% fetal bovine serum (Thermo Electron, Melbourne, Australia), 1% non-essential amino acids (FUJIFILM Wako Pure Chemical Corp.), and 30 µg/mL kanamycin (FUJIFILM Wako Pure Chemical Corp.). HuH-7 cells were cultured in DMEM with 1.0 g/L glucose (FUJIFILM Wako Pure Chemical Corp.) containing 10% fetal bovine serum and 30 µg/mL kanamycin. They were maintained at 37 °C in a 5% CO_2_-humified air atmosphere and subcultured every four days.

For cell growth and cytotoxicity assays (Section 4.2.7), the cells were seeded into a 96-well microplate at a density of 15,000 cells/cm^2^ and cultured overnight. For the lipid micelle-induced ApoB-48 secretion experiments (Section 4.2.8), the Caco-2 cells were seeded in 12-well Transwell plates with a polycarbonate microporous membrane (0.4 µm pore size, area of 1.12 cm^2^; Corning, Inc., Corning, NY, USA) at a density of 40,000 cells/cm^2^. After 24 h of seeding, the cell culture medium in both the apical and basolateral chambers was refreshed every two days to create a model system of an intestinal monolayer with a functional tight junction. After three weeks of culturing, transepithelial electrical resistance (TEER) was determined as a measure of cell monolayer integrity using an epithelial volt-ohm meter Millicell ERS-2 (EMS Millipore Corp., Burlington, MA, USA). Cell monolayers with a TEER value greater than 600 Ω cm^2^ were used.

#### 4.2.7. Cell Growth and Cytotoxicity Assay

Matoa peel extract prepared earlier (Section 4.1.2) was resuspended in dimethyl sulfoxide at a concentration of 100 mg/mL and used as a stock fraction. Before application, it was diluted in fresh culture medium to generate the final concentrations required. Next, the culture medium was removed from the cells on a 96-well plate, and the medium containing matoa peel extract was applied and incubated for an additional 24 h. The cell growth inhibitory activity and cytotoxicity of matoa peel extract was determined using Cell Counting Kit-8 (CCK-8; Dojindo Laboratories, Kumamoto, Japan) and the Cytotoxicity Lactate Dehydrogenase Assay Kit-WST (Dojindo Laboratories), respectively, according to the manufacturer’s instructions. The percentage of cell growth inhibitory activity in the CCK-8 assay was calculated by comparing the value of the treated cells with that of the control cells. The percentage of LDH activity of the treated cells was calculated by comparing the value to the maximum LDH release (100%) in the control cells, to which the provided lysis buffer was added.

#### 4.2.8. Lipid Micelle-Dependent ApoB-48 Secretion in Caco-2 Monolayers

Lipid-micelle-containing serum-free DMEM was prepared according to the recipe of mixture #5, described by Chateau et al. [18]; it contained OA (600 µM), cholesterol (50 µM), 2-monooleoylglycerol (200 µM), lysophosphatidylcholine (200 µM), and sodium taurocholate (2.0 mM). Additionally, 2.0 mM sodium taurocholate-containing serum-free DMEM was prepared for the blank experiment. For incubation of Caco-2 monolayers with the aforementioned lipid-containing DMEM, the media in the basolateral wells were replaced with fresh medium (1.5 mL). The blank, control, and treatment group wells were prepared by replacing the apical well media with 0.5 mL of 2.0 mM sodium taurocholate-containing serum-free DMEM, 0.5 mL of freshly prepared lipid micelles, and matoa peel extract-containing lipid micelles, respectively. After 24 h of incubation at 37 °C, the culture medium in each basolateral well was collected and stored at −80 °C until use. The levels of ApoB-48 protein in the basolateral chamber were determined using an LBIS Human ApoB-48 ELISA Kit (FUJIFILM Wako Shibayagi Corp., Gunmma, Japan) according to the manufacturer’s instructions. Measurements were performed in duplicate and are representative of three independent experiments.

#### 4.2.9. OA-Dependent Lipid Accumulation in HuH-7 Cells

HuH-7 cells plated at a density of 25,000 cells/cm^2^ in a 12-well plate were allowed to grow overnight. The next day, the medium was replaced with serum-free DMEM containing 1% free fatty acid-free bovine serum albumin (FUJIFILM Wako Pure Chemical Corp.) in the presence or absence of 0.5 mM OA (Cayman Chemical, Ann Abor, MI, USA) and incubated further for 24 h. After OA treatment, the cells were fixed with 4% paraformaldehyde and stained with Oil Red O (FUJIFILM Wako Pure Chemical Corp.) for 20 min. After washing the cells with phosphate-buffered saline, the accumulated lipids in the cells that stained red were observed under a phase-contrast microscope (IX73; Olympus Corp., Tokyo, Japan).

#### 4.2.10. Statistical Analysis

Results are expressed as mean ± standard deviation. One-way analysis of variance followed by the Tukey-Kramer post-hoc test was used to assess significance. Dunnett’s test was performed to assess the effect of matoa peel extract on cell growth and toxicity. Microsoft Excel 2016 (Microsoft Corporation, Redmond, WA, USA) and MEPHAS web application software (http://www.gen-info.osaka-u.ac.jp/testdocs/tomocom/mokuji1-e.html, accessed on 3 November 2021) were used for the statistical analysis. Statistical significance was set at *p* < 0.05.

### 4.3. Chemical Analyses

#### 4.3.1. Identification of Compound **1** in MPP

Methanol (300 mL) was added to MPP (10.4 g) and sonicated for 40 min. The mixture was allowed to stand for 24 h at ambient temperature, filtered, and concentrated. The extract was mixed with a chloroform–methanol (1:1) solution (50 mL) and sodium sulfate (Na_2_SO_4_), filtered, and concentrated. Methanol (20 mL) was added to the residue and mixed using a ThermoMixer C (Eppendorf, Hamburg, Germany) at 40 °C for 20 min at 1000 rpm. The resulting suspension was centrifuged (2126 × *g*; 15 min), and the supernatants were combined and concentrated to obtain a crude methanolic extract (1.1 g). The methanolic extract (0.81 g) was solubilized with a 50% aqueous methanolic solution (16 mL) for 16 h at ambient temperature and centrifuged. The supernatant was filtered through a 0.22 µm polypropylene syringe filter (Membrane Solutions, LLC, Auburn, WA, USA) to prepare samples for high-performance liquid chromatography (HPLC). Preparative HPLC was performed using a JAI LC-9201 system with an ultraviolet detector (UV-254) and a refractive index detector (RI-50s) (Japan Analytical Industry Co., Ltd., Tokyo, Japan). A sample (8.0 mL) of the filtrate was injected into an aqueous size exclusion chromatography column (JAIGEL-GS320; 500 mm × 20 mm i.d.; JAI, Tokyo, Japan), which was eluted with a 50% aqueous methanol solution at a flow rate of 5.0 mL/min; the injection volume was 2.0–2.5 mL. The eluted fractions were collected and dried to yield compound **1** (14.2 mg; 0.4% yield). Compound **1** was identified by NMR spectroscopy and compared with data from the literature [19,23,38,39]. NMR spectra were recorded on an ECZ–600R (JEOL Ltd., Tokyo, Japan). The data for compound **1** are included in Figure S2. Methanol and chloroform were purchased from FUJIFILM Wako.

#### 4.3.2. Hydrolysis of Saponins and Extraction of Sapogenin (Hederagenin)

Hydrolysis of saponins was conducted according to previously described methods [40,41,42,43] with modifications. A sample of ca. 40 mg of dried peel powder of matoa or salak was weighed accurately and transferred to a 1.5 mL tube. Methanol (1 mL) was added, followed by mixing for 30 min at 2000 rpm and 40 °C using a ThermoMixer C (Eppendorf), sonication for 15 min, and centrifugation. For acid hydrolysis, the methanolic extract (1 mL) was transferred to a 5.0 mL tube and reacted with 20% (*w*/*w*) hydrochloric acid (1 mL) for 1 h at 1000 rpm and 60 °C using a ThermoMixer C. For the control (without acid hydrolysis), the extract (1 mL) was reacted with water (1 mL) instead of hydrochloric acid. The reaction mixture was extracted with chloroform (2 mL × 3) and concentrated. The crude extract was dissolved in methanol (1 mL) and evaporated under reduced pressure to remove the chloroform. The resulting residue containing sapogenin was dissolved in 1% tetrahydrofuran in methanol (1 mL), transferred to a 5-mL volumetric flask, and diluted with methanol to a total volume of 5 mL. The solution was filtered through a 0.22-µm nylon syringe filter (Shimadzu GLC Ltd.) to prepare samples for LC-MS. A hederagenin solution in methanol (0.96 µg/mL; 1 mL) was subjected to the same acid hydrolysis treatment and chloroform extraction to estimate the recovery rate. Hederagenin was obtained from TCI Chemicals (Tokyo, Japan). Hydrochloric acid and tetrahydrofuran (HPLC grade) were purchased from FUJIFILM Wako (Japan).

#### 4.3.3. HGS Content

Hederagenin concentrations of the acid hydrolyzed extracts of matoa peel and salak peel (Section 4.3.2) were measured by LC-MS according to a previously described method [44] with modifications. HPLC was performed on a LC-20A Prominence system equipped with an SIL-20AC autosampler (Shimadzu, Japan). An LCMS-2020 mass spectrometer equipped with an electrospray ionization source operating in negative mode was used to identify and quantify the target analytes using chromatographic data processed using LabSolutions software (Shimadzu). Sample solutions (1 µL) were injected into an XBridge BEH C18 column (3.5 µm, 2.1 × 150 mm; Waters, Milford, MA, USA). The separation was achieved by applying a gradient elution of solvent A (10 mM ammonium bicarbonate) and B (methanol) as follows: 0–3 min, linear gradient 40–25% A; 3–18 min, 25% A; 18–30 min, 40% A. The flow rate was 0.2 mL/min, and the column temperature was 40 °C. The eluent was passed through an electrospray source. A capillary voltage of 3.5 kV was used in the negative ion mode. Nitrogen was used as the drying gas at a flow rate of 15 L/min and nebulizing gas at a flow rate of 1.5 L/min. The desolvation line temperature was set at 250 °C. The ion trap was operated in full scan mode from *m*/*z* 50 to 1000 and selected ion monitoring mode with *m*/*z* 471 for a molecular ion [M − H]^−^ of hederagenin. Identification and quantification were achieved by an external method using hederagenin standard solutions. Hederagenin (>98%; TCI Chemicals) was dissolved in a small amount of tetrahydrofuran and diluted with methanol to prepare the standard solutions. Quantification of the integrated peak areas of the samples allowed comparison with the calibration curves of the standard solutions. Samples from the acid hydrolyzed matoa solutions were subjected to further dilution before LC-MS injection. One milliliter of the solution was transferred to a 10-mL volumetric flask and diluted with methanol to a total volume of 10 mL to adjust its concentration within the range of the calibration curves used for quantification (10 ppm > conc. > 10 ppb). The HGS content in the samples was estimated by multiplying the molar concentration of hederagenin in the samples by the molecular weight (MW) of saponin (**1**). The MW for **1** was calculated as 883 g/mol based on the chemical formula for **1**: C_46_H_74_O_16_. The HGS content of the fruit peel on a dry weight basis was estimated by dividing the content of HGS in the samples by the weight of the dried peel samples. Methanol and ammonium bicarbonate were purchased from Sigma-Aldrich (Tokyo, Japan).

## 5. Conclusions

MPP decreased BW, visceral fat weight, hepatic lipid content, and serum TG levels in HFD-fed rats, possibly because of the HGS content of the peel powder. The in vitro data suggest that these anti-obesity effects of MPP were partly mediated by the inhibition of intestinal lipid absorption, as demonstrated using a Caco-2 monolayer. Therefore, MPP holds potential as a food ingredient with an anti-obesity effect, although a safety evaluation is still required.

## Figures and Tables

**Table 1:** Body, tissue, and organ weights and dietary intake in Sprague–Dawley rats fed four different controlled diets for four weeks (Animal Experiment 1).

Parameter	Experimental Group														
	N				HF				1M				1S		
Initial BW (g)	250	±	3 ^a^		251	±	6 ^a^		249	±	9 ^a^		247	±	4 ^a^
Final BW (g)	390	±	10 ^a^		442	±	22 ^b^		436	±	12 ^b^		442	±	33 ^b^
Dietary intake (g/day)	21.1	±	0.4 ^a^		20.4	±	0.4 ^a^		20.6	±	1.3 ^a^		19.8	±	1.8 ^a^
Energy intake (kJ/day)	342	±	6 ^a^		400	±	8 ^b^		403	±	25 ^b^		387	±	35 ^b^
Liver (g)	10.3	±	0.6 ^a^		12.3	±	1.1 ^b^		12.0	±	0.8 ^ab^		12.7	±	1.4 ^b^
Kidney (g)	2.8	±	0.1 ^a^		2.9	±	0.2 ^a^		2.9	±	0.4 ^a^		2.7	±	0.4 ^a^
Spleen (g)	0.8	±	0.1 ^a^		0.8	±	0.1 ^a^		0.8	±	0.1 ^a^		0.8	±	0.1 ^a^
Peritesticular fat (g)	5.1	±	1.2 ^a^		9.1	±	1.8 ^b^		8.5	±	1.0 ^b^		8.7	±	1.5 ^b^
Perirenal fat (g)	7.4	±	1.3 ^a^		11.9	±	2.7 ^b^		9.5	±	2.0 ^ab^		10.9	±	2.1 ^ab^
Mesenteric fat (g)	5.5	±	0.6 ^a^		8.9	±	1.1 ^b^		7.9	±	0.8 ^ab^		8.3	±	1.4 ^b^

BW, body weight; N, normal diet; HF, high-fat diet; 1M, high-fat diet containing 1% MPP; 1S, high-fat diet containing 1% SPP. Data are presented as means ± standard deviations (*n* = 6). Means in a row with different superscript letters are significantly different among groups (*p* < 0.05).

**Table 2:** Serum glucose and lipid levels and serum hepatic enzyme activities in rats fed four different controlled diets for four weeks (Animal Experiment 1).

Parameter	Experimental Group														
	N				HF				1M				1S		
Glucose (mmol/L)	11.0	±	2.7		10.6	±	1.8		10.9	±	3.8		13.6	±	3.2
TG (mmol/L)	0.88	±	0.13		1.04	±	0.12		0.98	±	0.17		0.98	±	0.23
TC (mmol/L)	1.37	±	0.15		1.23	±	0.21		1.17	±	0.19		1.25	±	0.13
HDL-C (mmol/L)	0.85	±	0.16		0.63	±	0.16		0.69	±	0.15		0.59	±	0.15
ALT (U/L)	15.0	±	0.8		14.7	±	1.4		14.5	±	1.8		15.7	±	2.4
AST (U/L)	55.2	±	5.7		55.5	±	5.6		50.3	±	7.1		54.2	±	7.5
γ-GTP (U/L)	<1				<1				<1				<1		

N, normal diet; HF, high-fat diet; 1M, high-fat diet containing 1% MPP; 1S, high-fat diet containing 1% SPP; TG, triacylglycerol; TC, total cholesterol; HDL-C, high-density lipoprotein cholesterol; ALT, alanine transaminase; AST, aspartate transaminase; γ-GTP, gamma-glutamyl transpeptidase; data are presented as means ± standard deviations (*n* = 6). No significant difference in serum glucose and lipid levels was observed among groups.

**Table 3:** Body weights, dietary intake, tissue weights, and fecal triacylglycerol levels in Sprague–Dawley rats fed four different controlled diets for four weeks (Animal Experiment 2).

Parameter	Experimental Group														
	N				HF				1M				3M		
Initial BW (g)	225	±	8 ^a^		223	±	6 ^a^		226	±	9 ^a^		225	±	6 ^a^
Final BW (g)	391	±	13 ^a^		424	±	26 ^b^		401	±	28 ^ab^		373	±	31 ^a^
Diet intake (g/day)	21.8	±	0.6 ^a^		19.8	±	1.7 ^b^		18.6	±	1.5 ^bc^		17.3	±	1.5 ^c^
Energy intake (kJ/day)	353	±	10 ^a^		387	±	34 ^b^		364	±	29 ^ab^		339	±	29 ^a^
Liver (g)	10.8	±	0.8 ^ab^		12.2	±	1.1 ^a^		11.2	±	1.5 ^ab^		10.1	±	1.4 ^b^
Kidney (g)	2.7	±	0.1 ^a^		2.8	±	0.2 ^a^		2.8	±	0.3 ^a^		2.6	±	0.2 ^a^
Spleen (g)	0.8	±	0.1 ^ab^		0.9	±	0.1 ^a^		0.8	±	0.1 ^b^		0.8	±	0.1 ^ab^
Peritesticular fat (g)	6.1	±	0.9 ^a^		7.5	±	1.5 ^a^		6.2	±	2.0 ^a^		5.0	±	2.6 ^a^
Perirenal fat (g)	8.4	±	1.3 ^a^		9.8	±	2.1 ^a^		7.5	±	3.3 ^ab^		5.0	±	2.3 ^b^
Mesenteric fat (g)	5.8	±	0.9 ^a^		7.5	±	1.4 ^a^		5.5	±	2.0 ^a^		3.9	±	1.9 ^b^
Feces, dry weight (g/day)	2.0	±	0.3 ^a^		2.0	±	0.4 ^a^		2.1	±	0.3 ^a^		2.0	±	0.1 ^a^
Fecal TG (mg/day)	0.80	±	0.6 ^a^		3.55	±	1.8 ^b^		3.68	±	1.8 ^b^		3.48	±	1.3 ^b^

BW, body weight; N, normal diet (*n* = 12); HF, high-fat diet (*n* = 8); 1M, high-fat diet containing 1% MPP (*n* = 8); 3M, high-fat diet containing 3% MPP (*n* = 8); TG, triacylglycerol. Data are presented as means ± standard deviations. Means in the same row with different superscript letters are significantly different among groups (*p* < 0.05).

**Table 4:** Levels of serum glucose, lipids, and hepatic enzyme activity in Sprague–Dawley rats fed four different controlled diets for four weeks (Animal Experiment 2).

Parameter	Experimental Group																
	N					HF					1M				3M		
Glucose (mmol/L)	10.6	±	2.6 ^a^				10.1	±	2.9 ^a^		9.1	±	2.4 ^a^		8.3	±	1.7 ^a^
TG (mmol/L)	1.27	±	0.41 ^a^				1.04	±	0.33 ^ab^		0.84	±	0.13 ^bc^		0.50	±	0.19 ^c^
TC (mmol/L)	1.44	±	0.21 ^a^				1.12	±	0.11 ^b^		1.30	±	0.23 ^ab^		1.32	±	0.23 ^ab^
HDL-C (mmol/L)	0.88	±	0.17 ^a^				0.58	±	0.08 ^b^		0.77	±	0.19 ^ab^		0.71	±	0.23 ^ab^
ALT (U/L)	14.1	±	2.6 ^a^				15.1	±	1.5 ^a^		18.0	±	5.0 ^a^		15.9	±	1.3 ^a^
AST (U/L)	57.1	±	10.3 ^a^				57.9	±	11.4 ^a^		66.4	±	29.3 ^a^		53.5	±	8.0 ^a^
γ-GTP (U/L)	<1					<1					<1				<1		

N, normal diet (*n* = 12); HF, high-fat diet (*n* = 8); 1M, high-fat diet containing 1% MPP (*n* = 8); 3M, high-fat diet containing 3% MPP (*n* = 8); TG, triacylglycerol; TC, total cholesterol; HDL-C, high-density lipoprotein cholesterol; ALT, alanine transaminase; AST, aspartate transaminase; γ-GTP, gamma-glutamyl transpeptidase. Data are presented as means ± standard deviations. Means in the same row with different superscript letters are significantly different (*p* < 0.05).

**Table 5:** Hederagenin saponin content in matoa and salak fruit peel.

Peel	HGS Content [% (*w*/*w*)]		
Matoa	1.41	±	0.039 ^a^
Salak	0.0154	±	0.0026 ^b^

Data are presented as means ± standard deviation (*n* = 3). Means with different superscript letters are significantly different (*p* < 0.001).

**Table 6:** The composition of various controlled diets fed to Sprague–Dawley rats for 4 weeks.

Parameter	Diet Group					
	Normal (N)		High-Fat (HF)	HF + 1% Matoa (1M)	HF + 3% Matoa (3M)	HF + 1% Salak (1S)
Energy (kJ/kg)	16,200		19,500	19,500	19,500	19,500
Ratio of energy-producing nutrients (kJ %)						
Protein	21		17	17	17	17
Carbohydrate	69		43	43	43	43
Fat	10		40	40	40	40
Ingredient (g/kg diet)						
Bovine milk casein	200		200	200	200	200
L-cystine	3		3	3	3	3
Corn starch	422.5		262.5	262.5	262.5	262.5
α-Corn starch	132		132	132	132	132
Sucrose	102.5		102.5	102.5	102.5	102.5
Soybean oil	25		25	25	25	25
Lard	20		180	180	180	180
*t*-Butylhydroquinone	0.005		0.005	0.005	0.005	0.005
Cellulose	50		50	40	20	40
AIN-93G Mineral mix	35		35	35	35	35
AIN-93 Vitamin mix with choline bitartrate	10		10	10	10	10
Matoa peel powder	0		0	10	30	0
Salak peel powder	0		0	0	0	10

## Data Availability

The data that support the findings of this study are available from the corresponding authors upon reasonable request.

## Supplementary Materials

The following are available online. Figure S1: Matoa peel extract does not suppress oleic acid (OA)-induced lipid accumulation in HuH-7 cells. Figure S2: Chemical structure and data for 3-*O*-[α-L-arabinofuranosyl(1→3)-α-L-rhamnopyranosyl(1→2)-α-L-arabinopyranosyl]hederagenin (**1**).

## Sample Availability

Samples of the compounds are not available from the authors.
