# Supplementary material for: Anti-Obesity Effects of Matoa (Pometia pinnata) Fruit Peel Powder in High-Fat Diet-Fed Rats
Source: Molecules. 2021 Nov 7;26(21):6733. doi: 10.3390/molecules26216733 (PMC8588254; doi:10.3390/molecules26216733)
Supplement: Supplementary file 1 [file molecules-26-06733-s001.zip › Figure S1_20211011.pptx]

## Slide 1
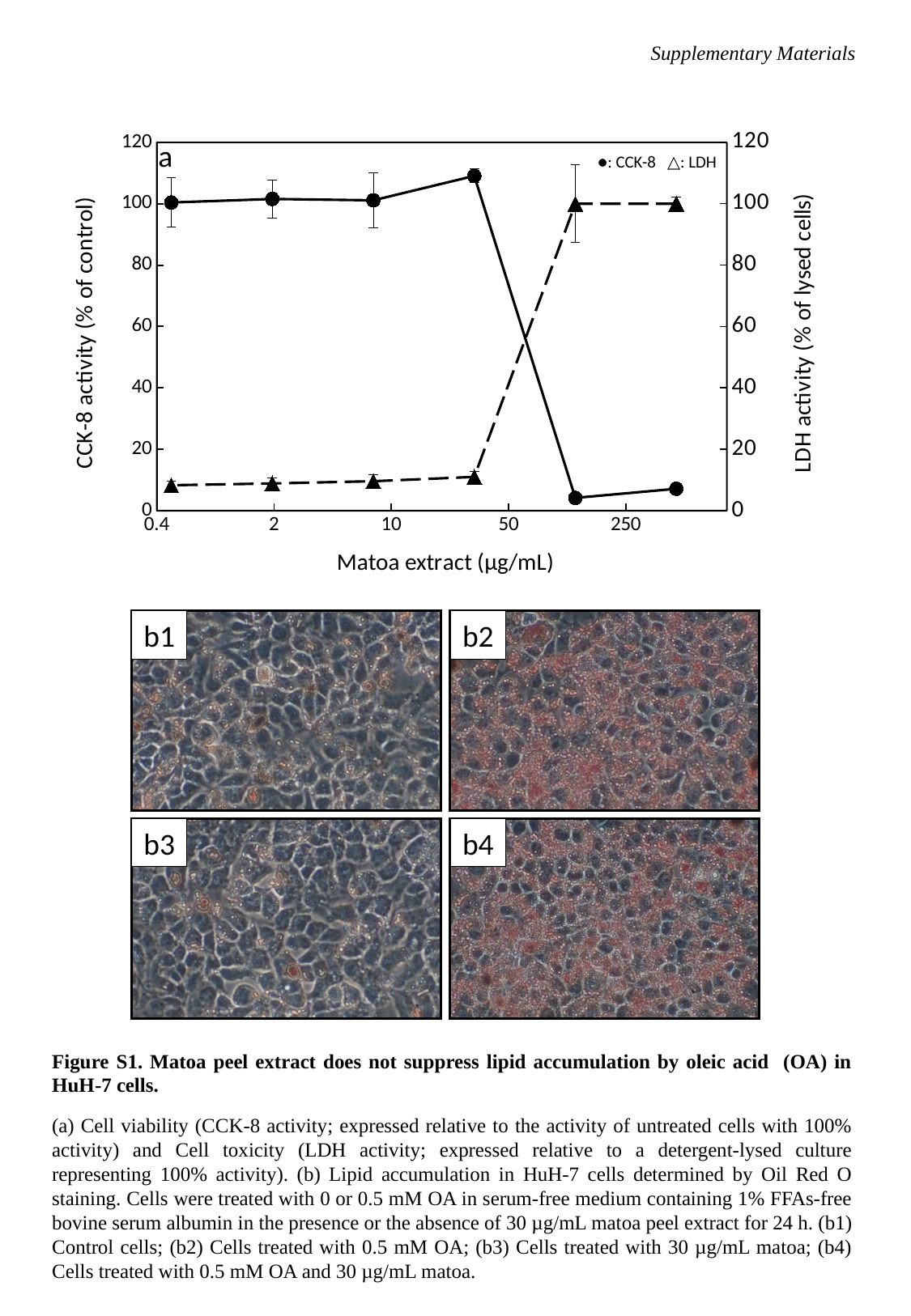

Supplementary Materials
### Chart
| Category | CCK-8 | |
|---|---|---|a
●: CCK-8 △: LDH
b1
b2
b3
b4
Figure S1. Matoa peel extract does not suppress lipid accumulation by oleic acid (OA) in HuH-7 cells.
(a) Cell viability (CCK-8 activity; expressed relative to the activity of untreated cells with 100% activity) and Cell toxicity (LDH activity; expressed relative to a detergent-lysed culture representing 100% activity). (b) Lipid accumulation in HuH-7 cells determined by Oil Red O staining. Cells were treated with 0 or 0.5 mM OA in serum-free medium containing 1% FFAs-free bovine serum albumin in the presence or the absence of 30 µg/mL matoa peel extract for 24 h. (b1) Control cells; (b2) Cells treated with 0.5 mM OA; (b3) Cells treated with 30 µg/mL matoa; (b4) Cells treated with 0.5 mM OA and 30 µg/mL matoa.
