# Supplementary material for: Anti-Obesity Effects of Matoa (Pometia pinnata) Fruit Peel Powder in High-Fat Diet-Fed Rats
Source: Molecules. 2021 Nov 7;26(21):6733. doi: 10.3390/molecules26216733 (PMC8588254; doi:10.3390/molecules26216733)
Supplement: Supplementary file 1 [file molecules-26-06733-s001.zip › Figure S2_20211011.pdf]

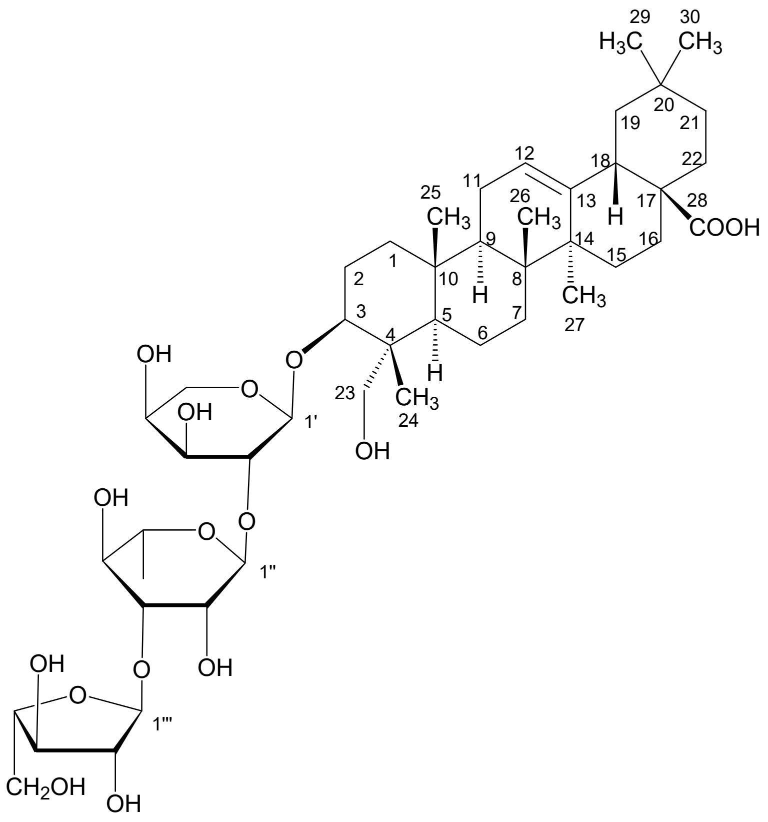

1

Figure S2. Chemical structure and data for 3-*O*-[α-L-arabinofuranosyl-(1→3)-α-L-rhamnopyranosyl-(1→2)-α-L-arabinopyranosyl]-hederagenin (**1**).

^{1}H NMR ($CD_3OD^{\dagger}$, 600 MHz): δ 0.70 (s, 3H, Me-24), 0.80 (s, 3H, Me-26), 0.90 (s, 3H, Me-29), 0.93 (s, 3H, Me-30), 0.96 (s, 3H, Me-25), 1.12 (m, 1H, H-19a), 1.16 (s, 3H, Me-27), 1.23 (d, *J*=6.6 Hz, Me-6''), 1.60 (m, 2H, H-1), 1.62 (m, 1H, H-9), 1.68 (t, *J*=13.2 Hz, H-19b), 1.72 (m, 1H, H-2a), 1.84 (m, 1H, H-2b), 1.89 (m, 2H, H-11), 2.83 (dd, *J*=3.6, 13.8 Hz, 1H, H-18), 3.31 (d, *J*=11.4 Hz, 1H, H-23a), 3.38 (dd, J=9.0, 9.6 Hz, 1H, H-4''), 3.58 (d, *J*=11.4 Hz, 1H, H-23b), 3.61 (m, 1H, H-3), 3.70 (m, 1H, H-2'), 3.70 (m, 1H, H-3''), 3.83 (m, 1H, H-5''), 3.84 (m, 1H, H-3'''), 3.92 (s, 1H, H-2''), 3.95 (br s, 1H, H-4'''), 3.51 (ddd, *J*=1.8, 5.4, 12.6 Hz, 1H, H-5'''a), 4.12 (d, *J*=2.4 Hz, 1H, H-2'''), 4.18 (ddd, J=3.6,

6.6, 8.4 Hz, 1H, H-4'), 4.49 (d, *J*=5.4 Hz, 1H, H-1'), 5.01 (d, *J*=1.8 Hz, 1H, H-1''), 5.09 (s, 1H, H-1'''), 5.23 (t, J=3.0 Hz, 1H, H-12).

^{13}C NMR (CD_3OD†, 150 MHz): δ 13.669 (CH_3, C-24), 16.379 (CH_3, C-25), 17.748 (CH_3, C-6''), 18.016 (CH_3, C-26), 18.772 (CH_2, C-6), 23.962 (CH_3, C-30), 24.048 (CH_2, C-16), 24.517 (CH_2, C-11), 26.442 (CH_3, C-27), 26.614 (CH_2, C2), 28.816 (CH_2, C16), 31.593 (C, C-20), 33.384 (CH_2, C-7), 33.556 (CH_3, C-29), 33.814 (CH_2, C-22), 34.877 (CH_2, C-21), 37.616(C, C-10), 39.665(CH_2, C-1), 40.498(C, C-8), 42.729 (CH, C-18), 42.968 (C, C-14), 43.964 (C, C-4), 47.210 (CH_2, C-19), 47.621 (C, C-17), 48.048 (CH, C-9), 49.5 (CH, C-5), 63.258 (CH_2, C-5'''), 64.435 (CH_2, C-23), 65.306 (CH_2, C-5'), 69.605 (CH, C-4'), 69.605 (CH, C-5''), 71.520 (CH, C-2''), 72.008 (CH, C-4''), 73.741 (CH, C-3'), 75.819 (CH, C-2'), 79.046 (CH, C-3'''), 79.381(CH, C-3''), 82.052, 82.895 (CH, C-2'''), 82.943 (CH, C-3), 86.935 (CH, C-4'''), 102.523 (CH, C-1''), 105.032 (CH, C-1'), 110.298 (CH, C-1'''), 123.626 (CH, C-12), 145.217 (C, C-13), 181.860 (C, C-28).

† NMR spectra were calibrated from solvent resonance (CH_3OH: 3.3 for ^{1}H; 49.0 for ^{13}C).
